# Relationship between blood heavy metals and female stress urinary incontinence from NHANES 2013–2018

**DOI:** 10.1265/ehpm.25-00021

**Published:** 2025-05-30

**Authors:** Yan-zhen Wu, Xi-meng Chen

**Affiliations:** 1 Department of gynecology and obstetrics, Wenzhou Central Hospital, Wenzhou 325000, Zhejiang, China; 2 Department of urology, Wenzhou Central Hospital, Wenzhou 325000, Zhejiang, China

**Keywords:** Female, Stress urinary incontinence, Heavy metals, Cholesterol

## Abstract

**Background:**

Research has demonstrated that heavy metals and cholesterol are associated with stress urinary incontinence (SUI) in women and that heavy metal exposure can cause dyslipidemia in humans. However, the potential mediating role of cholesterol in the relationship between heavy metals and female SUI remains unexplored.

**Methods:**

The study utilized data from the National Health and Nutrition Examination Survey database from 2013–2018. Blood lead (Pb), cadmium (Cd), total mercury (Hg), manganese (Mn), selenium (Se), and methyl mercury (MeHg) were included in the study. The single and combined effects of the six metals exposure on SUI were examined using logistic analysis, restricted cubic spline (RCS) curves, weighted quantile sum (WQS) regression, and bayesian kernel machine regression (BKMR). The mediating effects of total cholesterol (TC) and low-density lipoprotein cholesterol (LDL-C) were investigated through mediation analysis.

**Results:**

The study included 2241 females, with 42.66% experiencing SUI. Initial analysis of six heavy metals revealed the associations between MeHg, Pb, Cd, total Hg, and SUI (all *P* < 0.05). WQS regression identified that Cd, Se, and Pb were major contributors to the mixed effect causing SUI. BKMR results indicated a positive mixed effect between six heavy metals and SUI. TC partially mediated the relationship of Pb, MeHg, and total Hg with SUI, and LDL-C partially mediated the association of Pb with SUI (all *P* for mediation < 0.05).

**Conclusions:**

Blood heavy metal concentrations influence the development of female SUI, with blood cholesterol mediating the association between different heavy metals and SUI.

**Supplementary information:**

The online version contains supplementary material available at https://doi.org/10.1265/ehpm.25-00021.

## 1 Introduction

Stress urinary incontinence (SUI) is a common disorder of involuntary urine leakage from the external urethral orifice due to increased abdominal pressure from activities such as sneezing, coughing, and laughing, which occurs predominantly in females [1, 2]. In the United States, the overall prevalence of SUI (defined as any symptom in the previous year) among adult women is approximately 40–46% [3, 4]. Age, childbirth, and obesity are risk factors for the development of female SUI [5, 6]. Urinary incontinence significantly impacts women’s quality of life, often leading to embarrassment and inconvenience [7]. Consequently, investigating the influencing factors of SUI is essential to improve the health of women.

Certain heavy metals, including lead (Pb) and mercury (Hg), present substantial health hazards due to their persistence, difficult elimination, and bioaccumulation tendencies [8]. Previous studies indicate that heavy metals such as blood cadmium (Cd), Pb, and Hg may be associated with the development of female SUI [9]. Furthermore, exposure to a mixture of heavy metals such as selenium (Se), Pb, and Hg may influence blood total cholesterol (TC) and low-density lipoprotein cholesterol (LDL-C) levels in humans [10]. Cholesterol metabolites induce oxidative stress, which is associated with SUI pathology [11, 12]. In addition, cholesterol mediates the association of heavy metals with miscarriage, reproductive lifespan, and adolescent liver injury [13, 14]. However, the potential mediating role of cholesterol in heavy metals and the development of SUI remains unexplored.

To address this gap, the current study aimed to examine the relationship among blood heavy metal concentrations, cholesterol, and female SUI. The research utilized data from the National Health and Nutrition Examination Survey (NHANES) database, selecting variables such as demographic characteristics, blood heavy metal concentrations, and cholesterol related to SUI. The weighted quantile sum (WQS) regression and bayesian kernel machine regression (BKMR) were employed to investigate the mixed effect of blood heavy metals on the risk of SUI. Additionally, the study sought to determine whether cholesterol levels mediate the association between heavy metals and SUI.

## 2 Methods

### 2.1 Data sources and study population

The NHANES is sponsored by the Centers for Disease Control and Prevention’s National Center for Health Statistics. This survey assesses roughly 5,000 individuals of various ages annually across the United States, releasing updated information every two years in a cycle. NHANES is the only national health survey that includes health screenings, laboratory tests, and dietary interviews for participants of all ages (https://www.cdc.gov/nchs/nhanes/about/?CDC_AAref_Val=https:/www.cdc.gov/nchs/nhanes/about_nhanes.htm).

Three cycles (2013–2014 cycle, 2015–2016 cycle, 2017–2018 cycle) totaling 28,591 individuals surveyed from 2013–2018 were encompassed for this study. Following specific inclusion criteria, 2,241 females were ultimately included in the analysis. The inclusion criteria were: (1) Female patients aged ≥18 years with complete SUI information; (2) Individuals with complete blood heavy metals; and (3) Those with complete cholesterol information.

### 2.2 Determination of heavy metals and cholesterol

Blood Hg measurement involves using stannous chloride as a reducing agent and microwave digestion of blood samples to quantify inorganic mercury (InHg), ethyl mercury (EtHg), and methyl mercury (MeHg). Blood Pb, Cd, total Hg, manganese (Mn), and Se are directly quantified through mass spectrometry (MS) after sample dilution. To determine the concentrations of heavy metals in blood, the process begins by thoroughly mixing the blood sample through vortexing. A small portion of whole blood is then extracted from the patient sample to ensure an even distribution of cellular components. This extracted blood is subsequently diluted using a straightforward method: 1 part sample + 1 part water + 48 parts diluent. These dilutions are then employed for the direct measurement of Pb, Cd, total Hg, Mn, and Se in the whole blood samples using MS. The quality assurance and quality control protocols for heavy metals in this study include the following requirements: (1) The measured value of certified reference materials (STable 1) and spiked sample recovery (STable 2) need to be recorded for each batch of testing. (2) If the recovery falls outside the acceptable range (e.g., less than 80% or greater than 120%), recalibration or retesting is necessary. (3) Detection limits should be calculated based on CRM and blank samples (e.g., the limit of detection for blood Cd is approximately 0.1 ug/L). Detailed test methods are outlined in the NHANES Laboratory Procedure Manual document (https://wwwn.cdc.gov/nchs/nhanes/continuousnhanes/labmethods.aspx?BeginYear=2017). Samples were excluded from the analysis if the detection rate for blood heavy metals was below 75% [15], which was calculated using the following formula: detection rate = [(1 − number of cases below the lower limit of detection)/total number of detected cases] * 100%.

An enzymatic assay was employed to measure TC, while LDL-C was calculated based on TC, triglycerides, and high-density lipoprotein cholesterol (HDL-C) according to Friedewald: LDL-C = TC − HDL-C − triglycerides/5.

### 2.3 Outcome of study

The primary outcome of this study was SUI, which was identified by the question in the “Kidney Conditions-Urology” section of the questionnaire: “During the past 12 months, {have you/has SP} leaked or lost control of even a small amount of urine with an activity like coughing, lifting or exercise?”. A “Yes” response indicated SUI, while a “No” response indicated non-SUI. In this study, SUI was used as the dependent variable. NHANES professional diagnosis of disease and data collection through questionnaires. The use of this self-reported questionnaire for SUI assessment has been established as a reliable and valid method [16]. The questionnaire sections were all conducted by trained interviewers, allowing proxy interviewers and interpreters to answer when communication difficulties arose.

### 2.4 Covariates

In addition to the 8 heavy metal and 2 cholesterol variables, we also collected potential confounding variables [17, 18]: age, body mass index (BMI), poverty income ratio (PIR), race, education level (less than 9th grade; 9th grade or above), physical activity (PA), cotinine level, hypertension, and diabetes. Serum cotinine level, an indicator of smoking exposure (both active and passive), was measured using an isotope-dilution high-performance liquid chromatography/atmospheric pressure chemical ionization tandem mass spectrometry method. PA was calculated in metabolic equivalent (MET)-min/week by multiplying MET, weekly frequency, and duration of each activity. The activities considered in this study encompassed strenuous and moderate work-related activities, walking or bicycling, and strenuous and moderate leisure activities. Hypertension was defined as blood pressure ≥140 mmHg systolic and/or ≥90 mmHg diastolic, or the answer to the questionnaire “{Have you/Has SP} ever been told by a doctor or other health professional that {you/s/he} had hypertension, also called high blood pressure?” is “Yes”. Diabetes was defined when blood glucose glycosylated hemoglobin was ≥6.5% or self-reported diabetes based on the question “{Other than during pregnancy, {have you/has SP} ever been told by a doctor or health professional that {you have/{he/she/SP} has} diabetes or sugar diabetes?”.

### 2.5 Statistical analysis

Continuous but non-normally distributed data were described using median and quartiles (P_25_–P_75_), while categorical data were presented as frequencies and percentages. Comparisons between groups were conducted using the Mann-Whitney U test for quantitative data and the Chi-square test for categorical data. Univariate logistic analysis was utilized to initially explore the relationship between heavy metal concentrations as continuous data and SUI. Subsequently, due to the biased distribution of heavy metals in the human body, the heavy metal concentrations were further divided into three groups based on the tertiles, with the lowest tertile serving as a reference to analyze the relationship between heavy metal concentrations and SUI, including a trend test [19]. Subgroup analyses and interaction analyses were further performed by dividing the heavy metals into two groups (<25^th^ group and ≥25^th^ group) according to the 25^th^ quartile. Restricted cubic spline (RCS) curves were used to describe the nonlinear relationship between individual heavy metal exposure and SUI.

The combined effect of six heavy metals on SUI was evaluated using the WQS regression model, which also provided the contribution of each component to the overall effect. The BKMR model was employed to assess the mixed effect of combined exposure to heavy metals on SUI. We further calculated posterior inclusion probability (PIP), which assessed the contribution of the variable being included in the model, with larger values implying a greater contribution to the outcome. The single contamination model investigated the effect of individual metal concentrations on the outcome SUI, calculating the association of individual heavy metal exposure with the SUI at the 25th, 50th, and 75th percentiles while fixing the other heavy metals at the corresponding 25th, 50th, and 75th percentiles. The “est”, defined as the strength of the relation of individual heavy metal and SUI, was considered significant when its estimation was not zero. The univariate exposure-response relationship curves depicted the association between a single heavy metal exposure and SUI with other heavy metals fixed at the 50th percentile. To explore whether cholesterol levels mediated the relationship between heavy metal exposure and SUI, mediation analyses were performed using the “Mediation” package of the R software. All analyses were conducted using R (4.4.2) and *P* < 0.05 was set as statistically significant.

## 3 Results

### 3.1 The distribution of heavy metals

Table 1 presented the lower limit of detection, detection rate and concentration distribution of blood heavy metals of the participants in this survey. The LLOD of eight heavy metals were blood Pb (0.07 ug/dL), blood Cd (0.10 ug/L), blood total Hg (0.28 ug/L), blood Se (24.48 ug/L), blood Mn (0.99 ug/L), blood MeHg (0.12 ug/L), blood EtHg (0.16 ug/L) and blood inorganic Hg (0.27 ug/L). The detection rates of blood Pb, blood Cd, blood total Hg, blood Se, blood Mn, and blood MeHg were higher than 75%, whereas blood EtHg (0.71%) and blood InHg (23.03%) had lower detection rates (<75%). Therefore, InHg and EtHg were excluded from further analysis.

**Table 1:** The related information of blood heavy metals among survey participants.

**Variable**	**LLOD**	**Detection rate (%)**	**Mean**	**Selected percentiles**		

				**25th**	**50th**	**75th**
Blood Pb, ug/dL	0.07	100.00	1.07	0.53	0.85	1.33
Blood Cd, ug/L	0.10	95.98	0.53	0.21	0.34	0.59
Blood total Hg, ug/L	0.28	87.55	1.42	0.40	0.75	1.52
Blood Se, ug/L	24.48	100.00	191.81	175.07	189.16	205.65
Blood Mn, ug/L	0.99	100.00	10.76	8.04	10.05	12.74
Blood MeHg, ug/L	0.12	93.13	1.20	0.18	0.53	1.24
Blood EtHg, ug/L	0.16	0.71	-	-	-	-
Blood InHg, ug/L	0.27	23.03	-	-	-	-

*Abbreviations: LLOD, lower limit of detection; MeHg, methyl mercury; EtHg, ethyl mercury; InHg, inorganic mercury; Pb, lead; Hg, mercury; Cd, cadmium; Se, selenium; Mn, manganese.

### 3.2 Baseline characteristics

This study recruited 2241 female participants, including 956 with SUI (42.66%) and 1285 without SUI (57.34%). The median age of 2241 females was 51.00 years old. As shown in Table 2, age, BMI, race, hypertension, and diabetes were significant differences in the SUI group and non-SUI group (all *P* < 0.05). In the SUI group, the median age (55.00 vs. 47.00) and BMI (30.00 vs. 27.80) were higher than in the non-SUI group. Also, 49.19% of SUI patients suffered from hypertension and 19.21% of SUI patients suffered from diabetes, which was higher than those in the non-SUI group. In addition, the SUI group exhibited higher levels of TC (191.00 vs. 185.00), LDL-C (111.00 vs. 107.00), blood Pb (0.92 vs. 0.81), and blood Cd (0.37 vs. 0.32) compared to the non-SUI group (all *P* < 0.05).

**Table 2:** Characteristics of the study population.

**Variables**	**Total** **(n = 2241)**	**non-SUI** **(n = 1285)**	**SUI** **(n = 956)**	***P*-value**
Age, years	51.00[36.00,64.00]	47.00[32.00,63.00]	55.00[42.00,66.00]	<0.001
BMI, kg/m^2^	28.90[24.40,34.50]	27.80[23.400,33.60]	30.00[25.90,35.70]	<0.001
PIR	2.06[1.12,3.95]	2.10[1.14,3.91]	1.99[1.10,4.00]	0.848
Race, n (%)				<0.001
Hispanic	592(26.42)	330(25.68)	262(27.41)	
Non-Hispanic White	824(36.77)	400(31.13)	424(44.35)	
Non-Hispanic Black	468(20.88)	327(25.45)	141(14.75)	
Others	357(15.93)	228(17.74)	129(13.49)	
Education level (%, ≥9th grade)	2045(91.25)	1180(91.83)	865(90.48)	0.264
Hypertension, n (%)	923(42.61)	465(37.65)	458(49.19)	<0.001
Diabetes, n (%)	337(15.50)	161(12.80)	176(19.21)	<0.001
Physical activity (%, Active)	449(20.05)	248(19.30)	201(21.07)	0.301
Cotinine, ng/mL	0.03[0.01,0.38]	0.03[0.01,0.34]	0.03[0.01,0.57]	0.614
TC, mg/dL	188.00[164.00,215.00]	185.00[163.00,213.00]	191.00[165.00,218.00]	0.004
LDL-C, mg/dL	109.00[88.00,133.00]	107.00[88.00,130.00]	111.00[88.00,136.00]	0.039
Blood MeHg, ug/L	0.53[0.18,1.24]	0.53[0.18,1.30]	0.53[0.18,1.08]	0.362
Blood Pb, ug/dL	0.85[0.530,1.33]	0.81[0.50,1.30]	0.92[0.57,1.39]	<0.001
Blood Cd, ug/L	0.34[0.210,0.59]	0.32[0.20,0.57]	0.37[0.23,0.63]	<0.001
Blood total Hg, ug/L	0.75[0.400,1.52]	0.76[0.390,1.59]	0.73[0.41,1.39]	0.398
Blood Se, ug/L	189.16[175.07,205.63]	188.49[174.52,204.93]	190.15[176.01,206.50]	0.055
Blood Mn, ug/L	10.05[8.04,12.74]	10.06[7.99,12.68]	10.03[8.12,12.75]	0.685

*Abbreviations: BMI, body mass index; PIR, poverty income ratio; TC, total cholesterol; LDL-C, low-density lipoprotein cholesterol; MeHg, methyl mercury; Pb, lead; Hg, mercury; Cd, cadmium; Se, selenium; Mn, manganese.

### 3.3 Association between single heavy metals and female SUI

The results of univariate logistic regression revealed that blood MeHg, blood total Hg, blood Pb, and blood Cd were associated with SUI when heavy metal concentrations were continuous variables. Blood MeHg [OR(95%CI) = 0.95(0.911,0.992), *P* = 0.026] and blood total Hg [OR(95%CI) = 0.95(0.914,0.992), *P* = 0.025] were protective factors for the prevalence of SUI, and blood Pb [OR(95%CI) = 1.13(1.022,1.247), *P* = 0.018], blood Cd [OR(95%CI) = 1.36(1.173,1.576), *P* < 0.001] were risk factors for the prevalence of SUI. However, only Pb, Cd, and total Hg were associated with SUI when heavy metal concentrations were categorical variables based on the tertiles (Table 3).

**Table 3:** Univariate logistic analysis of six heavy metals and SUI.

**Variable**	**Odds Ratio**	**95%CI**	***P*-value**
As continuous variable			
Blood MeHg, ug/L	0.95	[0.911,0.992]	0.026
Blood Pb, ug/dL	1.13	[1.022,1.247]	0.018
Blood Cd, ug/L	1.36	[1.173,1.576]	<0.001
Blood total Hg, ug/L	0.95	[0.914,0.992]	0.025
Blood Se, ug/L	1.00	[1.000,1.006]	0.050
Blood Mn, ug/L	1.00	[0.983,1.025]	0.711
As categorical variable			
Blood MeHg, ug/L			
0.08–0.29	*Ref.*		
0.30–0.92	1.17	[0.958,1.440]	0.123
0.93–46.30	0.84	[0.687,1.038]	0.107
*P* for trend			0.114
Blood Pb, ug/dL			
0.07–0.62	*Ref.*		
0.63–1.15	1.24	[1.012,1.528]	0.038
1.16–14.26	1.43	[1.163,1.757]	0.001
*P* for trend			0.001
Blood Cd, ug/L			
0.07–0.25	*Ref.*		
0.26–0.47	1.23	[1.003,1.514]	0.047
0.48–7.50	1.41	[1.146,1.724]	0.001
*P* for trend			0.001
Blood total Hg, ug/L			
0.20–0.51	*Ref.*		
0.52–1.16	1.31	[1.067,1.606]	0.010
1.17–51.03	0.90	[0.734,1.109]	0.329
*P* for trend			0.348
Blood Se, ug/L			
85.15–180.00	*Ref.*		
180.05–199.27	1.18	[0.960,1.448]	0.116
199.29–453.62	1.15	[0.939,1.417]	0.173
*P* for trend			0.174
Blood Mn, ug/L			
2.91–8.78	*Ref.*		
8.79–11.60	1.04	[0.844,1.272]	0.737
11.61–52.00	1.07	[0.869,1.310]	0.535
*P* for trend			0.535

*Abbreviations: MeHg, methyl mercury; Pb, lead; Hg, mercury; Cd, cadmium; Se, selenium; Mn, manganese.

**Fig. 1:**
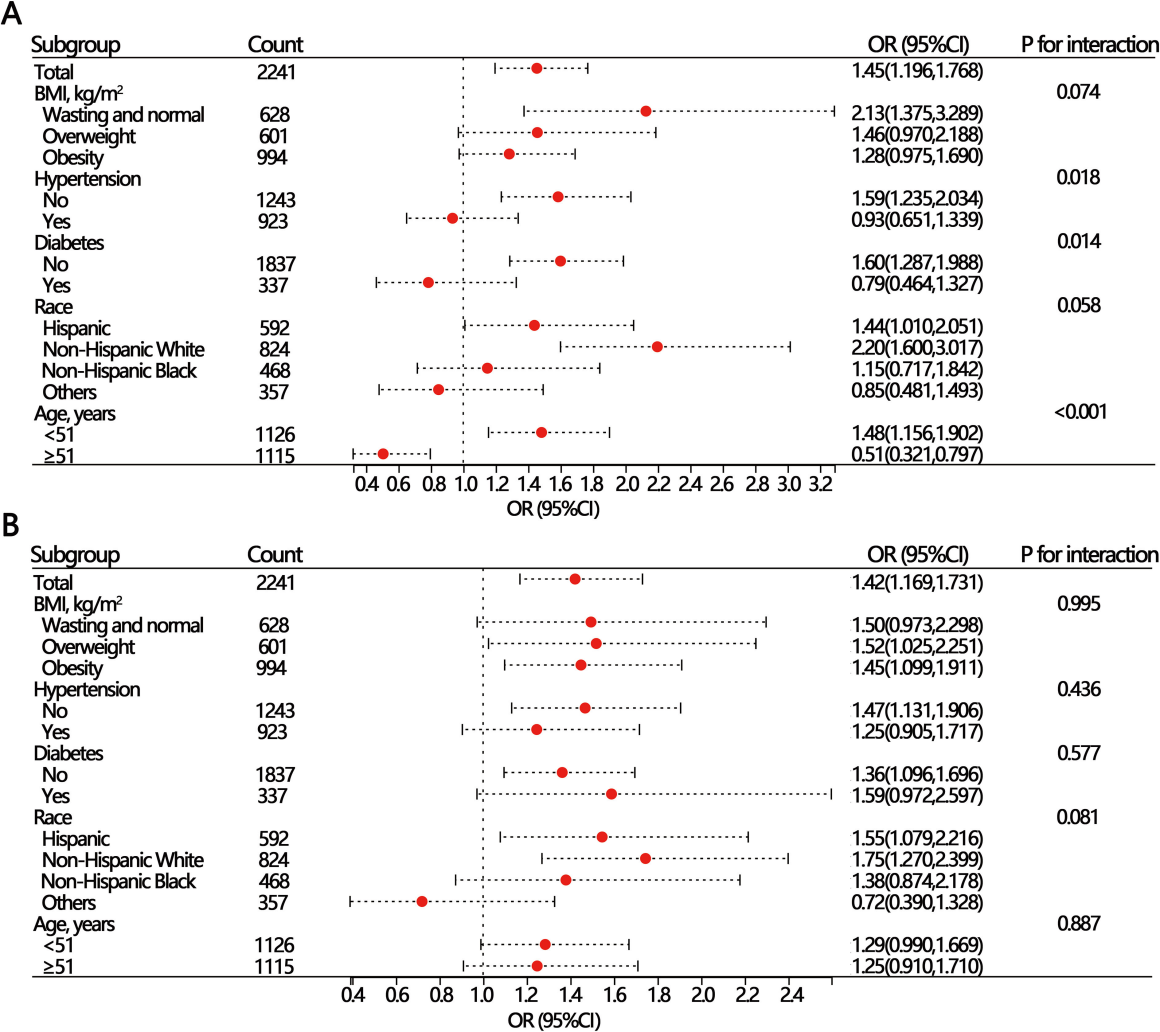
Subgroup and mediation analyses of blood Pb (A) and blood Cd (B).

We further divided the six heavy metals into two groups (<25^th^ group and ≥25^th^ group) according to the 25^th^ quartile and performed subgroup analyses as well as interaction analyses. The results indicated that only blood Pb and blood Cd were associated with SUI when the <25^th^ group was used as the reference group (Fig. 1A and Fig. 1B). Interaction analysis revealed that blood Pb was the only metal interacting with age, hypertension, and diabetes (Fig. 1A). The association between blood Pb and SUI was statistically significant in individuals without diabetes [OR(95%CI) = 1.60(1.287,1.988)] or without hypertension [OR(95%CI) = 1.59(1.235,2.034)], whereas it was not statistically significant in the diabetes or hypertension groups. Furthermore, the relationship between blood Pb and SUI was reversed in the different age subgroups. For individuals aged under 55, blood Pb was a risk factor for SUI [OR(95%CI) = 1.48(1.156,1.902)], but a protective factor in aged ≥55 [OR(95%CI) = 0.51(0.321,0.797)]. The interaction analyses for the other five heavy metals were not statistically significant (Fig. 1B, SFig. 1A, SFig. 1B, SFig. 2A, and SFig. 2B).

**Fig. 2:**
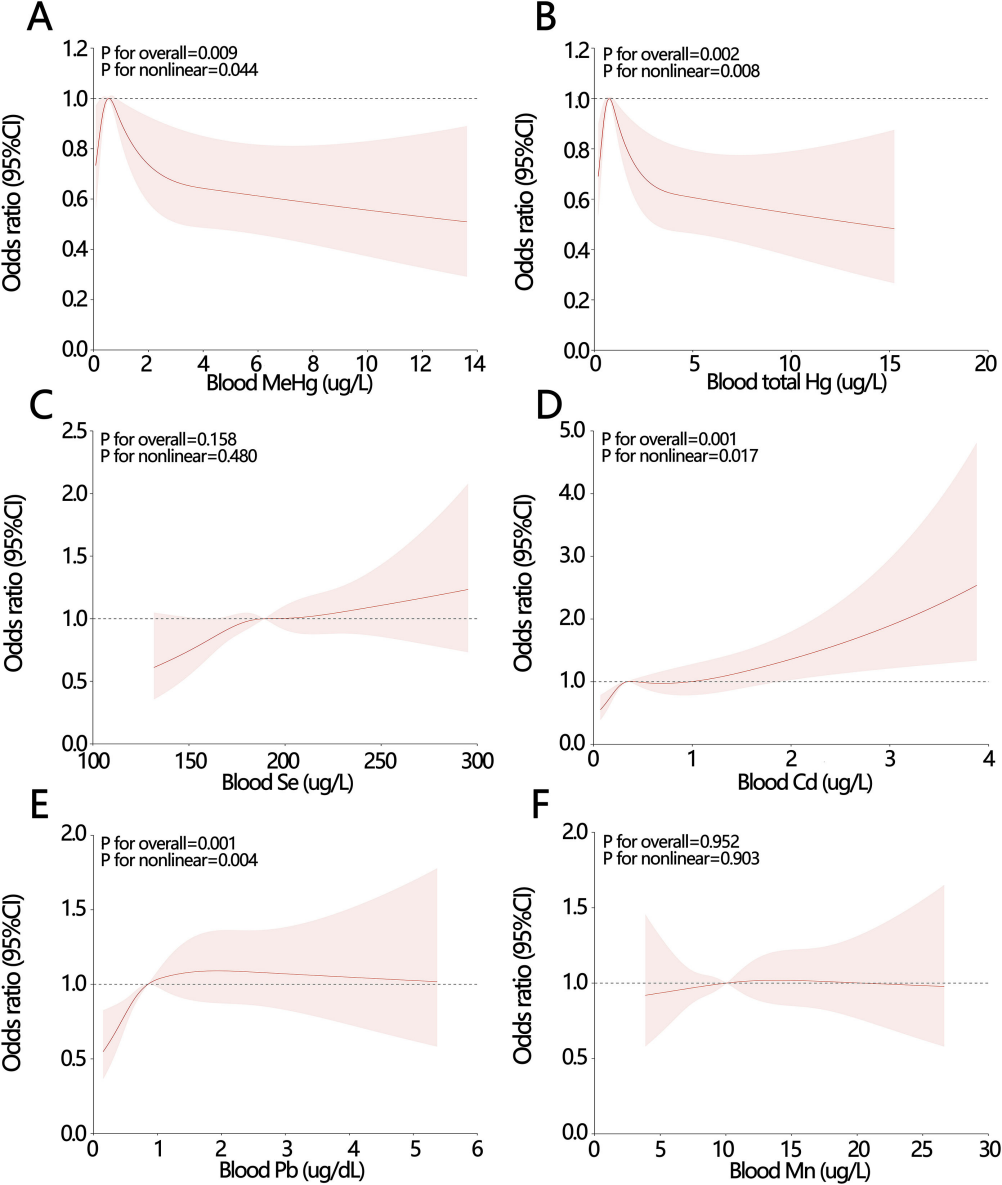
Restricted cubic spline (RCS) curves of six heavy metals on female stress urinary incontinence (SUI). (A) RCS curve between MeHg and SUI. (B) RCS curve between blood total Hg and SUI. (C) RCS curve between blood Se and SUI. (D) RCS curve between blood Cd and SUI. (E) RCS curve between blood Pb and SUI. (F) RCS curve between blood Mn and SUI.

The RCS analyses demonstrated that blood MeHg, blood total Hg, blood Cd, and blood Pb were significantly associated with the risk of SUI (Fig. 2A, Fig. 2B, Fig. 2D, and Fig. 2E). In contrast, blood Se and blood Mn were not associated with the risk of SUI (Fig. 2C and Fig. 2F). These findings were consistent with the results of the univariate logistic analysis for continuous heavy metal variables, with MeHg, Pb, Cd, and total Hg exhibiting nonlinear relationships with SUI. The associations between MeHg, total Hg, Cd, and SUI were J-shaped, while Pb and SUI displayed an inverted U-shaped relationship.

### 3.4 Mixed effects of heavy metals on female SUI

**Fig. 3:**
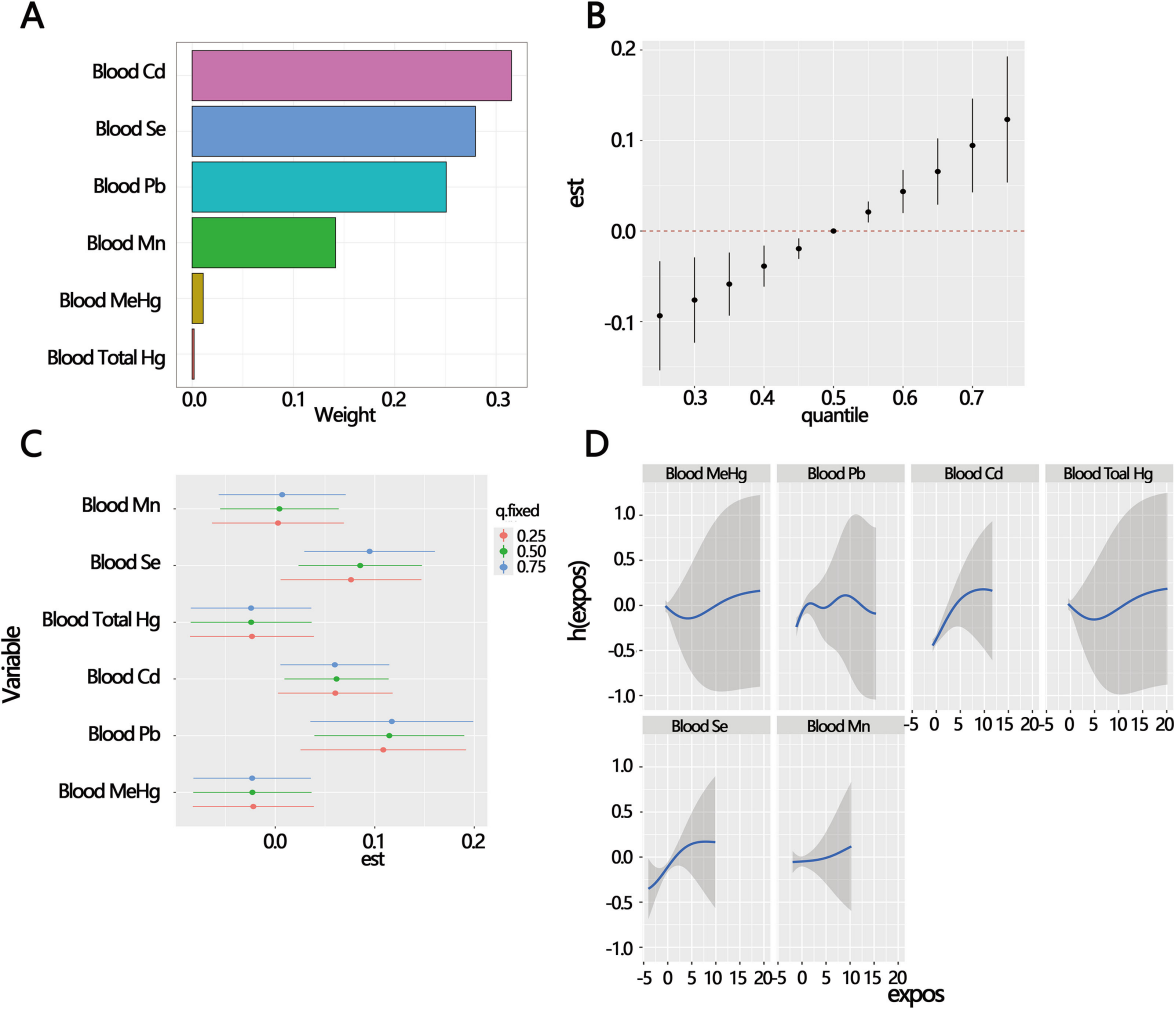
Mixed effects of co-exposure to mixed heavy metals on female stress urinary incontinence (SUI). (A) Weighted quantile sum (WQS) model regression index weights for blood heavy metals and SUI. (B) Mixed effects of the metal as a mixture on SUI by bayesian kernel machine regression (BKMR) model. (C) Single-contamination model between single heavy metal and SUI by BKMR. (D) Univariate exposure-response functions and 95% confidence intervals of single heavy metal by BKMR.

The WQS regression results indicated that among the mixed effects causing the risk of female SUI, blood Cd had the highest weight, followed by blood Se and blood Pb (Fig. 3A). Higher weights signified a greater contribution to the mixed effect. The six heavy metals were included in the BKMR model to explore the relationship between the heavy metals and female SUI. The results of BKMR revealed a positive mixed effect between the six heavy metals and female SUI (Fig. 3B). The PIP ranking for the six heavy metals was as follows: blood Se (0.960), blood Pb (0.908), blood Cd (0.890), blood MeHg (0.706), blood total Hg (0.638), and blood Mn (0.522) (Table 4). The results of WQS regression and PIP suggested that blood Se, blood Pb, and blood Cd played a primary role in the mixed effect of six heavy metals. The BKMR single contamination model revealed that there was a significant association between blood Se, blood Cd, blood Pb, and female SUI (Fig. 3C). The other blood heavy metals were set to their 50th percentile exposures to assess the individual effects function of the univariate heavy metal exposure (Fig. 3D).

**Table 4:** The posterior inclusion probability for six heavy metals.

**Variables**	**PIP**
Blood Se	0.960
Blood Pb	0.908
Blood Cd	0.890
Blood MeHg	0.706
Blood total Hg	0.638
Blood Mn	0.522

*Abbreviations: PIP, posterior inclusion probability; MeHg, methyl mercury; Pb, lead; Hg, mercury; Cd, cadmium; Se, selenium; Mn, manganese.

### 3.5 Mediation between heavy metals and female SUI

**Fig. 4:**
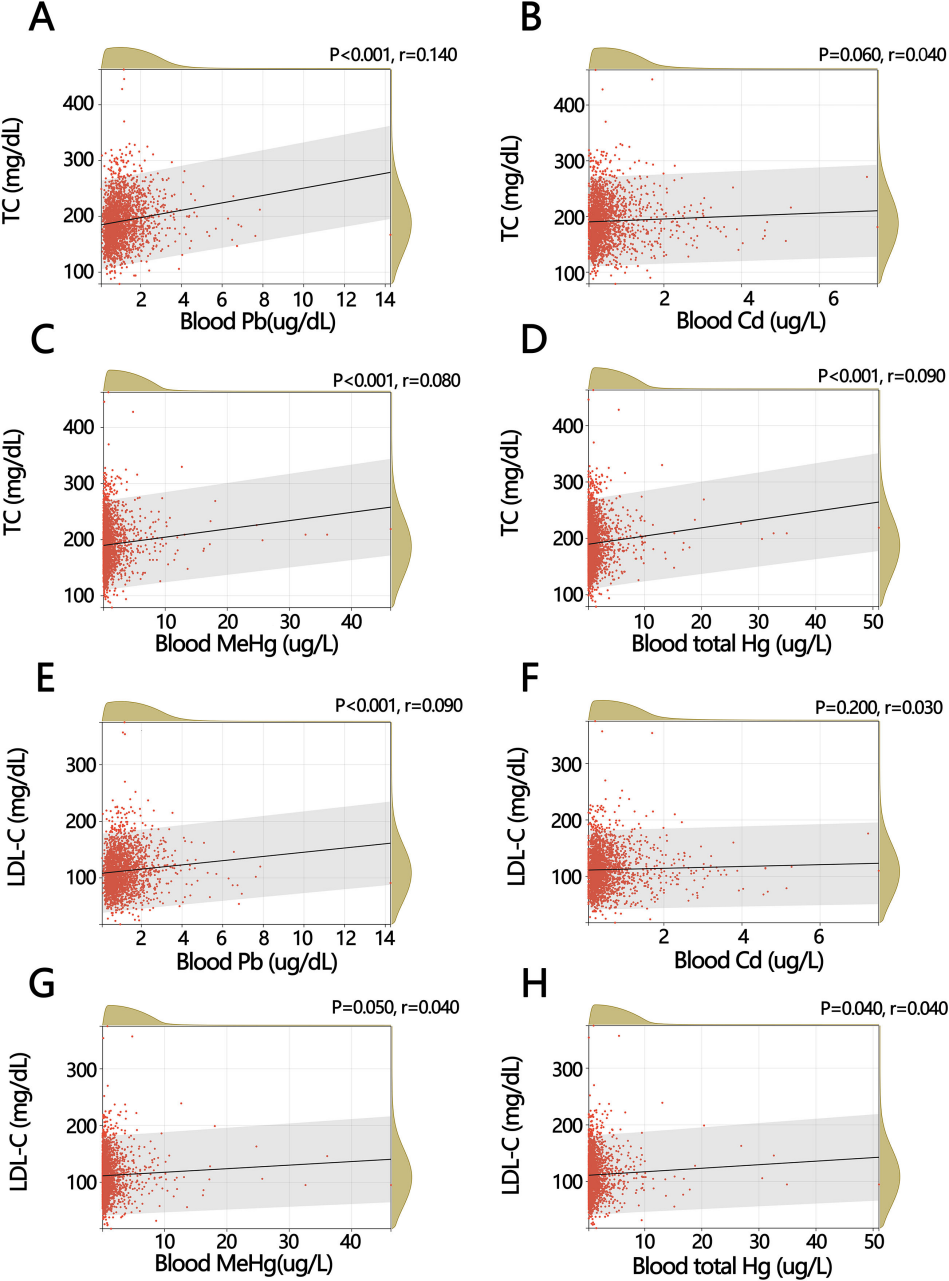
Correlation of total cholesterol (TC) and low-density lipoprotein cholesterol (LDL-C) with heavy metals. (A) The relationship between blood Pb and TC in the NHANES database, 2013–2018. (B) The relationship between blood Cd and TC. (C) The relationship between blood MeHg and TC. (D) The relationship between blood total Hg and TC. (E) The relationship between blood Pb and LDL-C. (F) The relationship between blood Cd and LDL-C. (G) The relationship between blood MeHg and LDL-C. (H) The relationship between blood total Hg and LDL-C.

Due to the potential for heavy metals to increase cholesterol levels, we explored the relationship between TC, LDL-C, and four heavy metals based on the results of univariate logistic regression (Fig. 4A–Fig. 4H). The results of correlation analysis showed that blood Pb (r = 0.140, *P* < 0.001), blood MeHg (r = 0.080, *P* < 0.001) and blood total Hg (r = 0.090, *P* < 0.001) were significantly positively correlated with TC (Fig. 4A, Fig. 4C and Fig. 4D), while blood Pb (r = 0.090, *P* < 0.001) and blood total Hg (r = 0.040, *P* = 0.040) were also positively correlated with LDL-C (Fig. 4E and Fig. 4H).

**Fig. 5:**
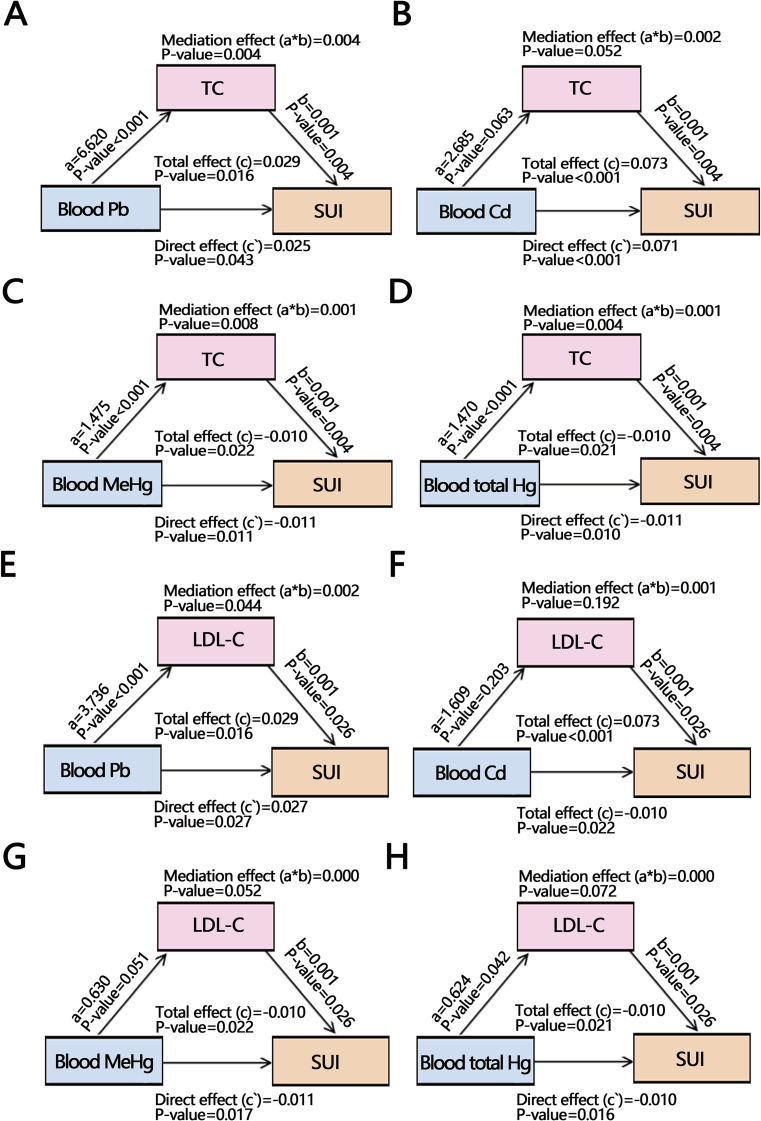
Mediation analysis of total cholesterol (TC) and low-density lipoprotein cholesterol (LDL-C) between heavy metals and stress urinary incontinence (SUI). (A) The mediation of TC between Blood Pb and SUI. (B) The mediation of TC between blood Cd and SUI. (C) The mediation of TC between blood MeHg and SUI. (D) The mediation of TC between blood total Hg and SUI. (E) The mediation of LDL-C between Blood Pb and SUI. (F) The mediation of LDL-C between blood Cd and SUI. (G) The mediation of LDL-C between blood MeHg and SUI. (H) The mediation of LDL-C between blood total Hg and SUI.

The results of the above analyses indicated that heavy metals were correlated with SUI. TC and LDL-C were related to some heavy metals, so we performed a mediation analysis (Fig. 5A–Fig. 5H). The mediation analysis of heavy metals, cholesterol, and SUI showed that TC and LDL-C partially mediated the association of Pb on SUI (Fig. 5A and Fig. 5E). Blood Pb could directly influence SUI or through TC/LDL-C indirectly influence SUI. For Cd and SUI, no mediation by TC and LDL-C was observed (Fig. 5B and Fig. 5F), indicating a direct effect of Cd on SUI without involving TC or LDL-C. In the relationship between MeHg or total Hg and SUI, TC played a partial mediating role (Fig. 5C and Fig. 5D), while LDL-C had no mediating effect (Fig. 5G and Fig. 5H), suggesting that MeHg or total Hg can affect SUI both directly and through TC modulation.

## 4 Discussion

SUI is a common disorder in women that seriously influences patients’ quality of life [20]. Previous research demonstrates that heavy metals and cholesterol are associated with female SUI and that heavy metal exposure can cause dyslipidemia in humans [21–23]. However, whether cholesterol mediates the relationship between heavy metals and female SUI has not been investigated. This study first confirmed the relationship between heavy metals and SUI, revealing non-linear associations between MeHg, Pb, Cd, total Hg, and SUI. The results of WQS and BMKR indicated that there was a positive joint effect between the six heavy metals and SUI, with Cd, Se, and Pb playing a dominant role.

Cd, Hg, and Pb are known to be toxic at very low concentrations, without a threshold for toxicity in organisms [24]. Previous research shows that Hg is positively correlated with the occurrence of SUI [9], whereas the present study found an inverse J-shaped correlation between MeHg or total Hg and SUI. The relationship between MeHg or total Hg concentration and SUI showed an initial increase in OR value followed by a decrease, consistently remaining below 1. The WQS results found that MeHg or total Hg contributed less to the mixed effect of causing SUI. However, Se had a more obvious dominant role in the mixed exposure model. Experimental evidence suggests an antagonism between Hg and Se, with Se potentially protecting against Hg through selenoprotein synthesis [25, 26]. Recent findings suggest that Hg and Se binding leads to the withdrawal of Se from selenoprotein synthesis and that Hg poisoning is essentially manifested through Se depletion [27]. Therefore, the complex impact of Se and Hg on SUI may be attributed to their interaction. Se is a crucial trace element that plays an essential role in various physiological processes in the body, such as oxidative stress response, inflammation regulation, apoptosis, and lipid metabolism [28]. However, elevated Se levels have been found to increase hepatic Selenoprotein P, which decreases tyrosine phosphorylation of insulin receptors in liver cells and reduces serine phosphorylation in muscle cells, thereby impairing insulin signaling and glucose metabolism, potentially leading to type 2 diabetes [29]. Diabetes mellitus is an independent risk factor for urinary incontinence in women, and blood glucose and glycated haemoglobin levels are positively associated with the severity of SUI [30]. An animal experiment demonstrates that excessive Se levels induce oxidative stress leading to dysregulation of mitochondrial homeostasis [31]. The incidence of SUI has been associated with mitochondrial homeostasis following oxidative stress of pelvic floor fibrous connective tissue dysregulation [32].

Cd and Pb are widely recognized toxic heavy metals affecting human health, lacking physiological functions but capable of causing severe adverse effects on various tissues, including the nervous system, liver, and kidney [33]. The results of our study showed that blood Pb and blood Cd were associated with the development of SUI in women, which is consistent with the findings of Ni et al. [34]. Both Cd and Pb induce increased levels of oxidative stress and apoptosis in vivo, and oxidative stress such as endoplasmic reticulum stress and apoptosis are associated with the development of SUI [35, 36]. Cd-induced oxidative stress may activate immune cells, potentially leading to elevated pro-inflammatory cytokine concentrations in exposed individuals [37]. Pb also plays an important role in the formation and development of inflammation in vivo, where it can act on gene expression levels and the synthesis of pro-inflammatory proteins [38], and inflammatory effects may similarly contribute to the occurrence of SUI [35, 36]. Cd and Pb, common endocrine disruptors, can impair the hypothalamic-pituitary-gonadal axis, indirectly inhibiting estrogen synthesis [39–41]. Reduced estrogen levels may result in decreased collagen content in the urethral epithelium, diminished α-adrenergic receptor sensitivity in the transverse sphincter, and reduced urethral closure, leading to SUI [42]. Animal study shows that Cd-induced inflammation impairs skeletal muscle function in mice [43], and Pb exposure inhibits myofibroblast differentiation and impaired the development and health of skeletal muscle [44], whereas further studies are needed to determine whether Cd or Pb impairs pelvic floor and urinary sphincter function, which are associated with the development of SUI. In addition, the present study found an inverted U-shaped relationship between Pb and SUI, which may be because people with high Pb exposure may develop other serious diseases (e.g., renal failure, hypertension) sooner, leading to a lower detection rate of SUI. Similarly, subgroup analyses revealed that the relationship of Pb on SUI varied across different age groups. In the lower age group, Pb was a risk factor for SUI, while a protective factor in the higher age group. This may be because older people exposed to lead may die earlier from cardiovascular or renal disease [45], and the detection rate of SUI may be underestimated by these competing risks., further validating the results of the RCS of Pb and SUI.

In a mediation analysis exploring whether TC and LDL-C impacted the development of heavy metal-induced SUI, we found that Pb was positively correlated with TC and LDL-C and that TC and LDL-C could partially mediate the correlation between Pb and SUI. Pb can significantly increase TC and LDL-C levels through oxidative stress, and cholesterol metabolism will also affect the development of SUI through oxidative stress [11, 12, 46]. Studies using animal models demonstrate that prolonged exposure to dyslipidemia may trigger epigenetic changes that impair the repair capacity of muscle-derived stem cells leading to SUI [47]. Furthermore, elevated LDL-C levels contribute to cholesterol accumulation on blood vessel walls and plaque formation, diminishing peripheral blood and oxygen supply [48]. Chronic ischemia and hypoxia of the pelvic floor muscles increase susceptibility to fatigue, ultimately resulting in impaired function causing SUI [49]. Additionally, TC mediated the association of MeHg and total Hg on SUI. Hg is linked to high TC [50, 51] and can promote lipid peroxidation by stimulating free radical production, interfering with antioxidant enzymes, and reducing bioavailable Se [52], potentially contributing to SUI development.

Our study has several strengths. Firstly, the present study initially investigated the role of cholesterol as a mediator in the relationship between heavy metals and SUI utilizing an extensive dataset from NHANES. Furthermore, we employed a variety of statistical methods such as WQS and BMKR to evaluate the combined effects of heavy metal exposure on female SUI. These comprehensive approaches enhance the reliability of our findings. However, there are also several limitations. The data, sourced from the NHANES database, may not be extrapolated due to racial constraints. Additionally, only blood heavy metal indicators were included in this study, and hence the relationship between urinary heavy metals and female SUI should be analyzed in the future to extend the exploration. Thirdly, the roles of Hg and Se were only discussed briefly, so future studies are needed to explore their mechanisms in depth. Lastly, concentrations of heavy metals in blood may reflect not only short-term exposure to heavy metals but also their long-term accumulation. Even if blood heavy metals reflect recent exposure, their pathogenic mechanisms may be the result of long-term cumulative effects. The association between current blood heavy metal concentrations and SUI over the past 12 months suggests a persistent effect of heavy metals. The actual long-term effects of exposure may be underestimated (as blood concentrations only partially reflect accumulation). SUI may range from transient episodes to lifelong persistence, and although the NHANES questionnaire emphasizes the occurrence of SUI “in the past 12 months”, which may have some questionnaire recall bias, it also investigates the frequency of SUI, as in the question “Less than once a month”, “A few times a month”, and so on. The cross-sectional data in this study do not allow us to determine the order of heavy metal exposure and SUI, so further cohort studies are needed to validate the results of this study.

## 5 Conclusion

There was a positive joint effect between the six heavy metals and female SUI, with Se, Pb, and Cd playing a dominant role in the overall effect of the heavy metal. Cholesterol mediated the impact of some heavy metals on SUI. Therefore, it is necessary to minimize exposure to heavy metals from potential environmental sources to prevent them directly or through modulation of lipid peroxidation inducing SUI. Due to the cross-sectional method of this study, additional cohort studies are required to validate these findings.

## Supplementary information

**Additional file 1:** **Supplementary Table 1.** Certified reference material for heavy metals in this study.

**Additional file 2:** **Supplementary Table 2.** The spiked recoveries of heavy metals in this study.

**Additional file 3:** **Supplementary Figure 1.** Subgroup and mediation analyses of blood Mn (A) and blood Se (B).

**Additional file 4:** **Supplementary Figure 2.** Subgroup and mediation analyses of blood MeHg (A) and blood total Hg (B).

## Declarations

### Data availability

Data will be made available on request.
