# Supplementary material for: Relationship between blood heavy metals and female stress urinary incontinence from NHANES 2013–2018
Source: Environ Health Prev Med. 2025 May 30;30:45. doi: 10.1265/ehpm.25-00021 (PMC12127084; doi:10.1265/ehpm.25-00021)
Supplement: Supplementary file 1 — Additional file 1: Supplementary Table 1. Certified reference material for heavy metals in this study. [file ehpm-30-045-s001.docx]

**Supplementary Table 1.** Certified reference material for heavy metals in this study.

| **Heavy metals** | **Sample** | **CRM** |
| --- | --- | --- |
| **Pb** | Whole blood | NIST SRM 955c (Toxic Elements in Caprine Blood) |
| **Cd** | Whole blood | NIST SRM 955c |
| **Mn** | Whole blood | NIST SRM 955c or Seronorm™ Trace Elements Whole Blood |
| **Total Hg** | Whole blood | NIST SRM 955c or IAEA-086 (Human Hair) |
| **Se** | Whole blood | NIST SRM 1598a (Human Serum) |
| **MeHg** | Whole blood | NIST SRM 1946 (Lake Superior Fish Tissue) |
| InHg | Whole blood | **NIST SRM 955c** (Toxic Elements in Caprine Blood) |
| **EtHg** | Whole blood | Custom Spiked Sample (labs are required to prepare their own thimerosal spiked blood samples since EtHg is not commercially available as a CRM) |

*Abbreviations: CRM, certified reference material; MeHg, methyl mercury; EtHg, ethyl mercury; InHg, inorganic mercury; Pb, lead; Hg, mercury; Cd, cadmium; Se, selenium; Mn, manganese.
