# Supplementary material for: Relationship between blood heavy metals and female stress urinary incontinence from NHANES 2013–2018
Source: Environ Health Prev Med. 2025 May 30;30:45. doi: 10.1265/ehpm.25-00021 (PMC12127084; doi:10.1265/ehpm.25-00021)
Supplement: Supplementary file 2 — Additional file 2: Supplementary Table 2. The spiked recoveries of heavy metals in this study. [file ehpm-30-045-s002.docx]

**Supplementary Table 2.** The spiked recoveries of heavy metals in this study.

| **Heavy metals** | **Spiked Recovery** | **Typical Recovery** |
| --- | --- | --- |
| **Pb** | 85–115% | 90–110% |
| **Cd** | 85–115% | 88–112% |
| **Mn** | 80–120% | 85–115% |
| **Total Hg** | 85–115% | 90–110% |
| **Se** | 80–120% | 85–115% |
| InHg | 85–115% | 88–110% |
| **MeHg** | 80–120% | 85–115% |
| **EtHg** | 75–125% | 70–110% |

*Abbreviations: MeHg, methyl mercury; EtHg, ethyl mercury; InHg, inorganic mercury; Pb, lead; Hg, mercury; Cd, cadmium; Se, selenium; Mn, manganese.
